# Identifying approaches for assessing methodological and reporting quality of systematic reviews: a descriptive study

**DOI:** 10.1186/s13643-017-0507-6

**Published:** 2017-06-19

**Authors:** Kusala Pussegoda, Lucy Turner, Chantelle Garritty, Alain Mayhew, Becky Skidmore, Adrienne Stevens, Isabelle Boutron, Rafael Sarkis-Onofre, Lise M. Bjerre, Asbjørn Hróbjartsson, Douglas G. Altman, David Moher

**Affiliations:** 1 Ottawa Methods Centre, Clinical Epidemiology Program, Centre for Practice-Changing Research, Ottawa Hospital Research Institute, Ottawa, Ontario Canada; 2 Translational Research in Biomedicine (TRIBE) Program, University of Split School of Medicine, Split, Croatia; 3 INSERM, UMR 1153, Centre of Research in Epidemiology and Statistics Sorbonne Paris Cité, University Paris Descartes, Paris, France; 4 Graduate Program in Dentistry, Federal University of Pelotas, Pelotas, RS Brazil; 5 Department of Family Medicine, University of Ottawa, Ottawa, Ontario Canada; 6 Bruyère Research Institute, Ottawa, Ontario Canada; 7 School of Epidemiology, Public Health and Preventive Medicine, University of Ottawa, Ottawa, Ontario Canada; 8 Center for Evidence-Based Medicine, University of Southern Denmark & Odense University Hospital, Odense, Denmark; 9 Centre for Statistics in Medicine, Nuffield Department of Orthopaedics, Rheumatology & Musculoskeletal Sciences, University of Oxford, Oxford, UK; 10 Centre for Journalology, Canadian EQUATOR Centre, Clinical Epidemiology Program, Centre for Practice-Changing Research, Ottawa Hospital Research Institute, Ottawa, Ontario Canada

**Keywords:** Reporting quality, Methodological quality, Systematic reviews, Guideline adherence

## Abstract

**Background:**

The methodological quality and completeness of reporting of the systematic reviews (SRs) is fundamental to optimal implementation of evidence-based health care and the reduction of research waste. Methods exist to appraise SRs yet little is known about how they are used in SRs or where there are potential gaps in research best-practice guidance materials.

The aims of this study are to identify reports assessing the methodological quality (MQ) and/or reporting quality (RQ) of a cohort of SRs and to assess their number, general characteristics, and approaches to ‘quality’ assessment over time.

**Methods:**

The Cochrane Library, MEDLINE®, and EMBASE® were searched from January 1990 to October 16, 2014, for reports assessing MQ and/or RQ of SRs. Title, abstract, and full-text screening of all reports were conducted independently by two reviewers. Reports assessing the MQ and/or RQ of a cohort of ten or more SRs of interventions were included. All results are reported as frequencies and percentages of reports.

**Results:**

Of 20,765 unique records retrieved, 1189 of them were reviewed for full-text review, of which 76 reports were included. Eight previously published approaches to assessing MQ or reporting guidelines used as proxy to assess RQ were used in 80% (61/76) of identified reports. These included two reporting guidelines (PRISMA and QUOROM) and five quality assessment tools (AMSTAR, R-AMSTAR, OQAQ, Mulrow, Sacks) and GRADE criteria. The remaining 24% (18/76) of reports developed their own criteria. PRISMA, OQAQ, and AMSTAR were the most commonly used published tools to assess MQ or RQ. In conjunction with other approaches, published tools were used in 29% (22/76) of reports, with 36% (8/22) assessing adherence to both PRISMA and AMSTAR criteria and 26% (6/22) using QUOROM and OQAQ.

**Conclusions:**

The methods used to assess quality of SRs are diverse, and none has become universally accepted. The most commonly used quality assessment tools are AMSTAR, OQAQ, and PRISMA. As new tools and guidelines are developed to improve both the MQ and RQ of SRs, authors of methodological studies are encouraged to put thoughtful consideration into the use of appropriate tools to assess quality and reporting.

**Electronic supplementary material:**

The online version of this article (doi:10.1186/s13643-017-0507-6) contains supplementary material, which is available to authorized users.

## Background

With the global annual expenditure of biomedical research estimated to be in excess of 100 billion USD [1], it is no surprise that the extent of published literature is growing each year, with PubMed® housing over 24 million citations, for example [2]. Researchers and decision makers have recognized that although there are hundreds of thousands of studies of healthcare interventions, the quality of research and reporting is variable. Evidence indicates that unless research is adequately designed and reported, the resources invested in research are not used effectively [1]. One estimate suggests that at least 50% of published research studies were poorly conducted making them difficult to interpret and use to inform best practice [1].

Systematic reviews (SRs) are considered the gold standard for healthcare decision-making as they evaluate the quality and confidence of all of the available evidence addressing specific questions, such as the benefits and harms of specific health care interventions. When SR conduct is optimal, that is, when best practices are employed to minimize biases in the process of collecting, appraising, and synthesizing the evidence, researchers can best understand whether or not they can be confident in the findings [3, 4]. Further, when SR reporting is optimal, the essential information is presented for practice guideline developers and other stakeholders, such as policy makers and clinicians to facilitate translation into guidance and improved patient care.

Criteria for assessing the quality of primary research emerged in the late 1980s with the rise of evidence-based medicine. This set the stage for guidelines assessing quality of SR conduct to be developed. Several sets of criteria had been developed early on including Mulrow [5] and Sacks criteria [6]. It was not until Oxman and Guyatt developed the Overview Quality Assessment Questionnaire (OQAQ) [7] in 1991, that a validated tool for assessing methodological quality (MQ) existed for SRs of intervention studies. More than a decade after OQAQ, A Measurement Tool to Assess Systematic Reviews (AMSTAR) [8] was developed and validated in 2007 to address additional SR quality criteria including potential sources of bias that were not included in the OQAQ tool. In 2010, AMSTAR was revised (R-AMSTAR) to provide a quantitative scoring method to assess quality [9]. With criteria available for assessing SR conduct, it was apparent that SR authors address the standards for improving reporting quality (RQ) as well. In 1999, the Quality of Reporting of Meta-analyses (QUOROM) statement [10] was created to evaluate the completeness of reporting of meta-analysis of randomized trials. Subsequently, in 2009, QUOROM was updated as the Preferred Reporting Items of Systematic reviews and Meta-Analyses (PRISMA) statement [11] to address several conceptual and methodological advances in the conduct and reporting of SRs. The development and adoption of SR MQ and RQ tools aim to assess, and hopefully improve, the design, conduct, and reporting of SRs. Which tools are accepted and used by SR authors to assess MQ and completeness of reporting was unclear.

We set out to identify methodological evaluations assessing the MQ and/or RQ of SRs published from 1990 to 2014 in order to determine the approaches that were used.

## Methods

### Definitions and important concepts

We defined SRs and meta-analyses in line with that provided by the Cochrane Collaboration and the PRISMA statement [12, 13]. We adopted the term ‘overview’, to mean a summary of evidence from more than one SR, including the combination of different populations, different interventions, different outcomes (both favourable ones and adverse events), or different conditions [14, 15]. It is synonymous with ‘systematic review of systematic reviews’, ‘reviews of reviews’, or an ‘umbrella review’. We have included publications of ‘methodological overviews’, meaning research that has assessed the MQ and/or RQ of a cohort of SRs and refer to these publications simply as ‘reports’.

#### Methodological quality and completeness of reporting

It is necessary to make clear the distinction between MQ and RQ. MQ addresses how well a SR was designed and conducted (e.g. literature search, selection criteria, pooling of data) [8]. RQ evaluates the description of the methodology and findings [11]. Moreover, to distinguish from MQ, the concept of risk of bias to assess primary studies is used to refer to systematic flaws or limitations in the design, conduct, or analysis of research that distort the findings [16]. Examples are the Cochrane Risk of Bias tool for randomized controlled trials [17], ROBINS-I for non-randomized studies [18], QUADAS-2 [19] for diagnostic studies, and ROBIS for SRs [16]).

### Objectives

The objectives of this study are to identify reports assessing the MQ and/or RQ of SRs and to assess their general characteristics and approaches used.

### Eligibility criteria

#### Inclusion criteria

We included any methodological report published between January 1990 and October 2014 whose stated primary intent was to assess the quality of methodology, reporting, or other self-identified quality indicator(s) of a cohort of SRs of interventions.

#### Exclusion criteria

We excluded reports of clinical interventions, whose primary intent was not to look at methodological quality or reporting and rather to summarise SR evidence for use in healthcare decision-making; reports assessing the quality of SRs of diagnostic, screening, etiological, or prognostic studies only; and evaluations of SRs that include study designs other than randomized controlled trials such as, narrative reviews, rapid reviews, network meta-analyses, and editorials. Reports in languages other than English were excluded due to budget constraints (Additional file 1) [20–31]. Reports assessing fewer than 10 SRs, those whose aim was to assess the reliability of an assessment tool, those assessing SRs in relation to one methodological characteristic (e.g. search strategy only), or those only assessing SRs with pooled estimates of effect, were also excluded.

### Search methods

An experienced information specialist developed and conducted an extensive search of the Cochrane Library, EMBASE®, and Ovid MEDLINE®, including In-Process & Other Non-Indexed Citations, from January 1990 to May 23, 2012. All searches were updated on October 16, 2014. Potentially eligible titles and/or abstracts were identified using a combination of subject headings (e.g. ‘Meta-Analysis as Topic’, ‘Quality Control’, ‘Checklist’) and key words (e.g. ‘umbrella review’, scoring, compliance) (see Additional file 2). A second senior information specialist peer reviewed prior to execution [32]. Additional reports eligible for inclusion were identified by members of the research team prior to the start of the project and used as ‘seed’ articles when developing the electronic search strategy [33–35].

### Screening

**Fig. 1:**
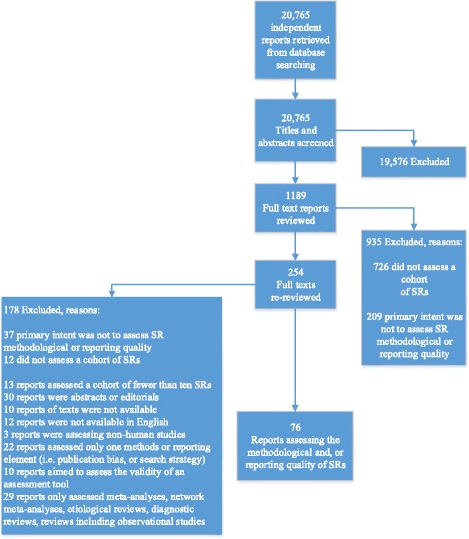
Flow of study reports

Titles and abstracts were screened for potentially inclusion using a liberal accelerated approach (i.e. one reviewer to include and two reviewers to exclude) [36]. Screening of full-text reports was completed independently in duplicate by a team of reviewers with experience in methodological reviews; 5% of potentially relevant articles were pilot tested. All screening disagreements were discussed, with any outstanding disagreements resolved by an independent third reviewer (DM). Data Management software, DistillerSR® [37], was used to manage retrieved records, screen reports, identify and track disagreements, and store data extracted. Results of the screening process are reported using a PRISMA flow diagram (Fig. 1).

### Data extraction

We developed standardized forms for data extraction of items of interest from the included reports. General characteristics and full data extraction was conducted by two reviewers in duplicate; a 10% random sample of reports was assessed for accuracy. A pre-extraction meeting was held for each extraction stage along with pilot testing to ensure consistency across reviewers. The following general characteristics were extracted: year of publication; number of included SRs; specified medical area; databases searched; language restrictions; SR definition; reporting of availability of study protocol; and source of funding. The method of assessing MQ or RQ of SRs was extracted. Additional items pertaining to the evaluated reviews were extracted including the following: types of publishing journals; Cochrane or non-Cochrane review; conflict of interest; number of SRs reported as updated reviews; number of SRs discussing limitations; critical appraisal of abstracts; number of SRs reporting meta-analysis; methods of meta-analysis used in the SRs (e.g. methods used for meta-analysis and type of measure, details of investigation of publication bias, whether or not heterogeneity was reported as assessed); whether interpretation were consistent with results; and whether a quantitative summary of quality was provided.

The attributes of primary of interest were to identify the method or tool used to assess (a) MQ of SRs (e.g. use of AMSTAR) and (b) RQ of SRs (e.g. use of PRISMA, identification of key methodological items). We classified tools/criteria into two groups: (1) items obtained from existing, published tools and (2) those developed by the report authors for their assessment.

Adherence data in relation to the MQ and RQ criteria were also extracted from those reports that provided it and are reported in a separate manuscript [38].

### Analyses

Summary statistics are reported as frequency and percentage of reports. No formal inferential statistical analyses were conducted. A post hoc decision was made to look at publications by their intent to assess MQ only, RQ only, or both MQ and RQ. This decision was made in order to identify all methods or tools used by overview authors to assess methodological conduct or reporting. In addition, we can identify whether the appropriate methods or tools were used to assess MQ or RQ of SRs. Differences in SR characteristics such as funding, limitations, and language restrictions whose intent was to assess MQ or both MQ and RQ can also be determined. This decision was made without prior review of the data by one of us (DM).

## Results

Of 20,765 unique title and abstract records retrieved and screened, 1189 full-text reports were reviewed for eligibility, of which 935 were excluded for not assessing a cohort of SRs or the primary intent was not to assess MQ or RQ. A secondary, full-text review of 254 remaining reports was carried out to ensure all exclusion criteria were met. A total of 76 reports were included (Fig. 1; see Additional file 3).

### Report characteristics

Characteristics of included reports are shown in Table 1. Of the 76 included reports, 66% (50/76) of them had a primary intent to assess MQ only, while the remaining one-third had a primary intent to assess either both MQ and RQ or RQ only; the latter two categories were grouped together, post hoc, given six reports (8%) assessed RQ only. Reports spanned a 21-year period; half were published between 2010 and 2014, indicating a marked increase in more recent years. A median of 51 SRs (interquartile range 25 to 105) were assessed in reports. SRs assessed were published within a specific medical field in 87% (66/76) of reports. Included SRs were reported to be of interventions in medical fields such as orthodontics, food and beverage, pediatrics, nephrology, and dermatology; there were no predominant fields. SRs were mainly from a general sample of reviews across medical journals; 7% (5/76) of reports evaluated a cohort of Cochrane reviews only. The majority of reports provided their source of SRs, whether via database searches or specific journals. Forty-one percent of reports did not report whether language restrictions were used, whereas the remaining 59% were nearly evenly divided as to whether they did or not. SR defined for inclusion criteria were provided by nearly 30%, while 43% used ‘systematic review’ as a search term, and 26% did not report this information. Few reports made reference to an available protocol. Thirty-nine percent of reports did not report the source of financial support for their research.

**Table 1:** Table of characteristics of reports presented by methodological quality or both methodological and reporting quality

Characteristic	Categorization	Reports assessing MQ only	Reports assessing MQ and RQ or RQ only	All reports
		*N* = 50, *n* (%)	*N* = 26, *n* (%)	*N* = 76, *n* (%)
Year of publication	1993–2009	26 (52)	11 (42)	37 (49)
	2010–2014	23 (46)	16 (62)	39 (51)
Number of assessed SRs	Median (IQR)	43 (21, 88)	68 (36, 109)	51 (25, 105)
	Range	10–327	10–487	10–487
Were SRs of particular medical field?	Yes	45 (90)	21 (81)	66 (87)
	No	5 (10)	5 (19)	10 (13)
Cohort of Cochrane SRs	Cochrane only	2 (4)	3 (12)	5 (7)
	Sample of reviews	23 (46)	10 (38)	33 (43)
	Specific journal sample or other	25 (50)	13 (50)	38 (50)
Number of databases searched	1	9 (18)	8 (31)	17 (22)
	2	5 (10)	2 (8)	7 (9)
	3	7 (14)	2 (8)	9 (12)
	4	7 (14)	5 (19)	12 (16)
	5	5 (10)	3 (12)	8 (11)
	6	2 (4)	2 (8)	4 (5)
	7	5 (10)	1 (4)	6 (8)
	8+	3 (6)	1 (4)	3 (4)
	Not reported	4 (8)	0 (0)	4 (5)
	Not applicable (select journals)	3 (6)	3 (12)	5 (7)
Reports restricting SRs by language	No restrictions	14 (28)	6 (23)	20 (26)
	Restricted to English	14 (28)	4 (15)	18 (24)
	Restricted to English and another specified languages	4 (8)	3 (12)	7 (9)
	Not reported	18 (36)	13 (50)	31 (41)
SR defined for inclusion criteria?	Yes, but no reference given	5 (10)	6 (23)	11 (15)
	‘Systematic review’ reported as a search term	21 (42)	12 (46)	33 (43)
	Cochrane collaboration and PRISMA Statement	4 (8)	5 (19)	9 (12)
	Other reference	3 (6)	0 (0)	3 (4)
	Not reported	17 (34)	3 (12)	20 (26)
Was a study protocol reported as available for this report?	Yes, link reported	1 (2)	2 (8)	3 (4)
	Yes, upon request	6 (12)	2 (8)	8 (11)
	No or not reported	43 (86)	22 (85)	65 (86)
Report Source of funding	Industry Funded	2 (4)	0 (0)	2 (3)
	Non-profit Funding	21 (42)	14 (54)	35 (46)
	Reported no funding	6 (12)	3 (12)	9 (12)
	Not reported	21 (42)	9 (35)	30 (39)

*MQ* methodological quality, *RQ* reporting quality

### Characteristics of SRs included in reports

Information reported by reports about included SRs is shown in Table 2. More than half (44/76) of reports reported information on the review’s source of funding, of which most (35/44) did so as part of their tool assessment. Conflict of interest information was reported in 45% (34/76) of reports in relation to a published tool assessment, whereas three reports did so as a stand-alone quality item. Heterogeneity assessment in reviews was considered as a marker for ‘quality’ in 62% (47/76) of reports, and 13% (10/76) reported this information as part of a published tool. Forty-two percent of reports stated how many of the included reviews had reported conducting a meta-analysis. Seventeen percent (13/76) reported which SRs were updates of an original review. Half of the reports extracted whether or not reviews considered issues of publication bias (formally or informally). Limitations were described in half (14/26) of the reports whose primary intent was to assess MQ and RQ, whereas only 4% (2/50) reported this information in reports whose intent was to assess MQ only. Critical appraisal of SR abstracts was reported in 29% (22/76) of reports, all of whose primary intent was to assess MQ and RQ. Thirty-eight percent of reports (29/76) gave consideration to how consistent review results were with review conclusions. A quantitative summary of SR quality across items or criteria were provided in 59% (45/76) of reports. The largest difference between reports with the intent to assess MQ only and MQ and RQ is the critical appraisal abstracts and limitations. This is likely attributed directly to the structure of RQ guidelines (e.g. PRISMA and QUOROM) specifically including reporting items for abstracts and limitations whereas MQ tools (e.g. OQAQ and AMSTAR) do not (Table 2).

**Table 2:** Information reported by reports about included SRs

Assessment of characteristics at the report level	Reports assessing MQ only	Reports assessing MQ and RQ or RQ only	All reports
	*N* = 50, *n* (%)	*N* = 26, *n* (%)	*N* = 76, *n* (%)
Source of funding	25 (50)	19 (73)	44 (58)
Conflict of interest	22 (44)	12 (46)	34 (45)
Heterogeneity investigated	32 (64)	15 (58)	47 (62)
Meta-analysis results reported	22 (44)	10 (38)	32 (42)
Updated reviews	6 (12)	7 (27)	13 (17)
Publication bias	25 (50)	13 (50)	38 (50)
Limitations discussed	2 (4)	14 (54)	16 (21)
Critically appraised abstracts	0 (0)	22 (85)	22 (29)
Interpretation consistent with results	21 (42)	8 (31)	29 (38)
Provided quantitative summary of quality or reporting across reviews	29 (58)	16 (62)	45 (59)

*MQ* methodological quality, *RQ* reporting quality

### Use of published assessment tools to assess quality over time

**Fig. 2:**
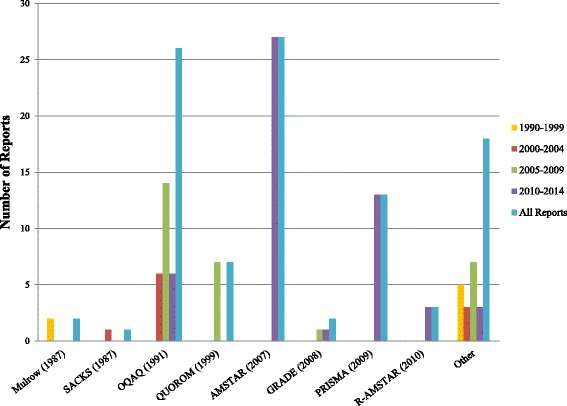
Tools or other criteria used by reports to assess SR quality or reporting over time

We assessed how frequently the published assessment tools were used across reports, in 5-year increments after 1999 (Fig. 2). For MQ, AMSTAR (27 reports) [8] and OQAQ (26 reports) [7] were used the most often in reports; others used R-AMSTAR (3 reports) [9], Mulrow criteria (2 reports) [5], Grading of Recommendations Assessment, Development, and Evaluation (GRADE) criteria (2 reports) [39], and Sacks criteria (1 report) [6]. We observed that although OQAQ (1991) use for MQ decreased after 2009, it was still being used despite the availability of AMSTAR as of 2007 (10 [after 2010] vs. 16 [before 2010]). For RQ, PRISMA (13 reports) [11] was used more often than its predecessor QUOROM (7 reports) [10]. No reports used QUOROM (1999) to assess RQ after PRISMA (2009) guidelines were published. In addition, several reports used their own criteria to assess quality after 2000, although OQAQ (1991) and QUOROM (1999) guidelines were available.

The above eight published tools were used across 80% (61/76) of reports. These reports used those tools alone, in combination with other tools or in combination with self-specified criteria. The remaining 15 reports used only self-specified criteria to assess quality.

### Published assessment tools used alone or in combination with other criteria to assess quality

**Fig. 3:**
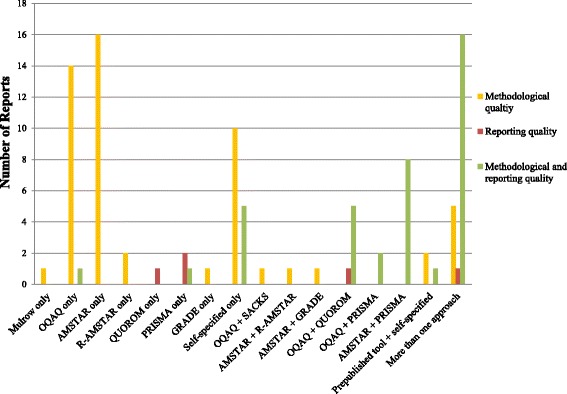
Published tools or self-specified criteria used alone or in conjunction presented by reported intent

Thirty-nine (51%) reports used published tools alone to assess quality (Fig. 3). Mulrow, GRADE, and QUOROM were used in one study each (1%; *n* = 76). AMSTAR and OQAQ were used the most frequently as stand-alone means to assess quality in 21% (16/76) and 20% (15/76) of reports, respectively. PRISMA and R-AMSTAR were used alone in three (4%; *n* = 76) and two (3%; *n* = 76) reports, respectively.

In 29% (22/76) of reports, published tools or criteria were used in conjunction with other criteria or tools to assess MQ or RQ (Fig. 3). Of those assessing MQ and RQ, 36% (8/22) used AMSTAR and PRISMA, 27% (6/22) of reports used OQAQ and QUOROM, and 9% (2/22) of reports used OQAQ and PRISMA. The remaining reports used a variety of combinations: AMSTAR and R-AMSTAR (5%; 1/22); OQAQ in conjunction with Sacks criteria (5%; 1/22); AMSTAR and GRADE (5%; 1/22); a published tool (OQAQ or Mulrow) in combination with self-specified criteria (9%; 2/22); and AMSTAR in conjunction with OQAQ and self-specified criteria (5%; 1/22). No reports evaluated a combined approach for assessing RQ. Due to the number of different combinations of tools and criteria used to assess quality in reports, it was not conducive to separate by time as well.

### Self-specified criteria to assess quality

Although OQAQ was published in 1991, authors developed their own criteria to assess MQ or RQ in 24% (18/76) of reports. Of these reports, 15 only used self-specified criteria to assess quality, and three reports used self-specified criteria in combination with another tool (as described above).

Quality assessment criteria used in these reports varied considerably. Furthermore, 13 reports used their own criteria to assess quality after the publication of both OQAQ (1991) and QUOROM (1999). Seven of the 18 reports (39%) did not provide any description of how they derived their quality assessment items. Of the remaining 11 reports, the majority were derived from the Oxman and Guyatt criteria. Two reports based criteria on Oxman and Guyatt, Jadad Scale (developed to assess primary studies), and QUOROM [40, 41]; one report on Oxman and Guyatt and the Jadad Scale [42]; two reports on Oxman and Guyatt and Mulrow [43, 44]; one report on Oxman and Guyatt, Light and Pillemer, and Mulrow [45]; one report used Oxman Guyatt and five additional criteria [46]; one report used Rapid Appraisal Protocol (RAP), National Center for the Dissemination of Rehabilitation Research (NCDRR) [47]; one report was based on PRISMA [48]; one on PRISMA and QUOROM [49]; and one report was based on Oxman and Guyatt, Hoving scale, discussion between three reviewers and expectations discussed from SRs by Sackett and Seers [50].

Only four of the 18 reports (22%) provided an explanation as to why they had created their own criteria. Two reports stated criteria was developed to evaluate quality of SRs in a specific medical field [40, 51]; one report [44] published in 1996 stated there was no gold standard for assessing quality; another report stated their quality assessment scale was developed to specifically evaluate patellofemoral pain syndrome [46].

### Appropriate use of tools to assess quality of conduct vs. reporting of conduct

We also assessed whether reports used the quality of methodological conduct (MQ) and reporting (RQ) tools appropriately. The majority of reports used the tools correctly (Fig. 3). However, we noted that several reports did not use the tools or criteria appropriately based on their reported or inferred intent. One report intended to assess both MQ and RQ but only used OQAQ criteria, a tool for assessing the quality of conduct. Another study intended to assess both MQ and RQ but only used PRISMA, a reporting guideline. Another report intended to assess RQ only and used both OQAQ and QUOROM which are tools used to assess quality of conduct and reporting of conduct. In addition, one report used GRADE to assess MQ.

## Discussion

We identified 76 reports in the health care literature assessing the MQ and/or the RQ of SRs published in the last 24 years in order to assess their quantity, characteristics, and methodology over time. The number of such reports increased over time with two-thirds intending to assess MQ only and the remaining 34% assessing either RQ only or both MQ and RQ. Although the number of reports increased, the criteria used to assess MQ and critical appraisal of SRs varied considerably across reports. Eight published tools were used in 80% of reports while review authors of the remaining reports only used their own criteria to assess quality. We identified PRISMA, AMSTAR, and OQAQ to be most commonly used tools.

This research parallels that of Dechartres et al. (2011), who investigated how quality is assessed in RCTs [52]. Those authors found great variety in how the quality of trials was assessed, from which the authors raised important issues about the tools and criteria that should be used to assess RCT methodological quality and reporting [53]. Although the diversity of assessment criteria and the number of scales used to assess RCT quality was greater, the authors found, as we did, that the number of methodological reviews had increased over time.

Our findings appear consistent with that of other research which suggests that tools used to assess MQ and RQ of SRs are variable [33, 54, 55]. In 2012, two studies were conducted to assess their methodological rigor [56, 57]. The first concluded that PRISMA, OQAQ, and AMSTAR were the most frequent methods of critical appraisal and quality assessment for SRs and that inconsistency in how SR quality is assessed should be reviewed [56]. The second identified at least nine methods of assessing SR quality and called for further empirical evidence to support the conduct of overviews [57].

In addition, despite lack of available evidence, it would be feasible to suggest that risk of bias assessment criteria at the trial level over time may influence trial conduct. By extension, critical appraisal criteria for SRs over time may in turn influence SR conduct. A small body of literature has started to emerge with regard to biases within SRs which would influence results of overviews [58]. The SR community currently lacks clear guidance regarding best SR practices to minimize biases. Standardized tools/criteria would provide the foundation upon which to develop more consistent critical appraisal criteria for SRs, which in turn could influence SR conduct.

Approximately 20% of methodological reports included in our investigation did not report their intention to assess either MQ or RQ in the title. This may be simply poor reporting or may highlight the general confusion over assessing SR ‘quality’ versus reporting of conduct. Quality of conduct (MQ) tools were developed to assess how well a SR was designed and conducted whereas reporting (RQ) guidelines were designed to guide SR authors in appropriate reporting of methodology and findings of SRs [8, 11]. The use of reporting guidance, such as PRISMA, to assess the methodological conduct or quality of SRs is not appropriate. While PRISMA serves as a resource to improve the quality of reporting of SRs, it is not an instrument to gauge the quality of a SR [59]. By extension, we also argue that the use of quality of conduct tools, such as OQAQ, to assess quality of reporting of SRs is not appropriate. While MQ criteria are important to improve quality of conduct, they do not assess quality of reporting [8]. Moreover, we also note that in one review, the authors inappropriately used GRADE and items from the Jadad Scale to assess MQ of SRs. GRADE was developed as a system for grading the quality of evidence of trials across studies for each important outcome, while the Jadad Scale was developed to assess the MQ/RQ of clinical trials assessing pain; thus, applicability to SRs is questionable [35, 60]. SR authors should adhere to MQ and RQ criteria to ensure high quality of conduct and accurate information is reported in SRs.

Methodologists focus on improving quality and reporting, and new tools and guidelines continue to be developed. For example, the US Institute of Medicine developed their own standards for assessing MQ in SRs and reporting [61]. Further, Cochrane recommends using the Methodological Expectations of Cochrane Intervention Review (MECIR) to guide conduct of Cochrane SRs for interventions [62]. Other recently published tools to improve quality in SRs include the Risk of Bias in Systematic Reviews (ROBIS) [16], developed to complement AMSTAR. The concept of risk of bias is distinct from MQ in that it assesses systemic flaws or limitations in the design, conduct, or analysis of research that distort the findings [16]. Although there is some content overlap between risk of bias and MQ criteria, the majority of criteria are distinct. For example, AMSTAR assesses whether at least two electronic sources were searched whereas ROBIS assesses whether the search included an appropriate range of databases for published and unpublished reports. Nonetheless, with the plethora of tools and guidance available, there remains confusion over the best criteria and tools to assess quality or reporting for consistent standards across reports. This may be simply due to SR authors being unaware of appropriate newer tools that exist; or tools or guidelines that have less criteria to assess are appealing simply due to lack of time; or they feel some criteria are lacking in the validated tools. Newer MQ and RQ tools such as AMSTAR and PRISMA were developed to reflect of the state of current SR methodology research. SR authors should put thoughtful consideration into use of appropriate MQ and RQ criteria to conduct their SR.

There are potential limitations to this study. All methodological research relating to the quality of studies, whether at the trial or SR level, is contingent upon the quality of reporting. In addition, due to feasibility, we have limited reports to English language only, reports assessing more than 10 SRs and reports using more than one methodology or reporting criteria to assess quality.

## Conclusions

In conclusion, a body of literature exists in evaluating the quality and reporting of SRs across a variety of medical fields. How quality is assessed varies and is similar to the conclusions in other reports. As new tools and guidelines are developed to improve both the MQ and RQ of SRs, SR authors are encouraged to give careful thought to the use of the most current and appropriate tools to assess quality and reporting as they reflect the state of current SR methodology research.

## Additional files

Additional file 1: List of non-English language studies [20–31]. (DOCX 15 kb)

Additional file 2: Search strategy. (DOCX 17 kb)

Additional file 3: List of included studies [33, 40, 42–51, 63–126]. (DOCX 22 kb)

## Abbreviations

AMSTAR: A Measurement Tool to Assess Systematic Reviews
GRADE: Grading of Recommendations Assessment, Development, and Evaluation
MECIR: Methodological Expectations of Cochrane Intervention Review
MQ: Methodological quality
OQAQ: Overview Quality Assessment Questionnaire
PRISMA: Preferred Reporting Items of Systematic reviews and Meta-Analyses
QUOROM: Quality of Reporting Of Meta-analyses
R-AMSTAR: Revised-A Measurement Tool to Assess Systematic Reviews
RQ: Reporting quality
SR: Systematic review
