# Supplementary material for: Identifying approaches for assessing methodological and reporting quality of systematic reviews: a descriptive study
Source: Syst Rev. 2017 Jun 19;6:117. doi: 10.1186/s13643-017-0507-6 (PMC5477124; doi:10.1186/s13643-017-0507-6)
Supplement: Supplementary file 1 — List of non-English language studies [20–31]. (DOCX 15 kb) [file 13643_2017_507_MOESM1_ESM.docx]

**Additional File 1. List of non-English Language Studies**

| Study | Title |
| --- | --- |
| Letelier 2014^20^ | Systematic reviews and metaanalysis: are the best evidence? |
| Grootens 2003^21^ | Increased number of systematic reviews in the Netherlands in the period 1991-2000 |
| Yan 2013^22^ | Publication and quality of systematic reviews/meta-analyses conducted by hospital pharmacists in China |
| Gonzalez de Dios 2012^23^ | Checklist in systematic reviews and meta-analysis: The PRISMA statement, beyond the QUOROM |
| Wang 2010^24^ | Quality assessment for chinese systematic reviews/meta-analyses in public health |
| Coenen 2013^25^ | Evaluating the methodologic quality of systematic reviews and meta-analyses. AMSTAR (A Measurement Tool for the Assessment of Multiple Systematic Reviews |
| Xu 2013^26^ | Methodological quality assessment of systematic reviews or meta-analyses of intervention published in the Chinese journal of evidence-based medicine |
| Wang 2014^27^ | A systematic assessment of the quality of systematic reviews/meta-analyses in radiofrequency ablation versus hepatic resection for small hepatocellular carcinoma |
| Morichon 2014^28^ | Taping: Trial by evidence? Review of systematic reviews |
| Liao 2012^29^ | Literature review report on efficacy evaluation about kudiezi injection |
| Chen 2012^30^ | Status quo analysis on TCM systematic reviews/meta-analyses published in Chinese journals |
| Jin 2012^31^ | Reporting and methodological quality of systematic reviews and meta-analyses in nursing field in China |
