# Supplementary material for: Identifying approaches for assessing methodological and reporting quality of systematic reviews: a descriptive study
Source: Syst Rev. 2017 Jun 19;6:117. doi: 10.1186/s13643-017-0507-6 (PMC5477124; doi:10.1186/s13643-017-0507-6)
Supplement: Supplementary file 2 — Search strategy. (DOCX 17 kb) [file 13643_2017_507_MOESM2_ESM.docx]

**Additional File 2. Search Strategy**

Database: Ovid MEDLINE(R) In-Process & Other Non-Indexed Citations and Ovid MEDLINE(R) <1946 to Present>, Embase <1980 to 2012 Week 20> Search Strategy:

--------------------------------------------------------------------------------

1 exp meta-analysis as topic/ (15767)

2 exp "Review Literature as Topic"/ (49807)

3 ((review$1 or overview$1) adj2 synthes$2).tw. (1832)

4 (umbrella review$1 or meta-review$1 or metareview$1).tw. (132)

5 ("review of reviews" or "reviews of reviews" or "overview of reviews" or "overviews of reviews" or "overview of overviews" or "overviews of overviews").tw. (637)

6 ("review of systematic reviews" or "reviews of systematic reviews" or "review of systematic overviews" or "reviews of systematic overviews" or "overview of systematic reviews" or "overviews of systematic reviews" or "systematic review of systematic reviews" or "systematic reviews of systematic reviews").tw. (444)

7 or/1-6 (67336)

8 exp Quality Control/ (249883)

9 exp Guideline Adherence/ (285655)

10 exp Checklist/ (5144)

11 st.fs. (494730)

12 (quality or standard* or criteri* or characteristic* or guideline* or guidance* or checklist* or check list* or score$1 or scoring).tw. (5398001)

13 (adher* or comply$ or compli$2 or complian* or conform* or evaluat* or assess*).tw. (7177120)

14 or/8-13 (10930935)

15 7 and 14 (15924)

16 limit 15 to yr="1990 -Current" (15558)

17 16 use prmz (8326)

18 exp "meta analysis (topic)"/ (3666)

19 exp "systematic review (topic)"/ (1851)

20 ((review$1 or overview$1) adj2 synthes$2).tw. (1832)

21 (umbrella review$1 or meta-review$1 or metareview$1).tw. (132)

22 ("review of reviews" or "reviews of reviews" or "overview of reviews" or "overviews of reviews" or "overview of overviews" or "overviews of overviews").tw. (637)

23 ("review of systematic reviews" or "reviews of systematic reviews" or "review of systematic overviews" or "reviews of systematic overviews" or "overview of systematic reviews" or "overviews of systematic reviews" or "systematic review of systematic reviews" or "systematic reviews of systematic reviews").tw. (444)

24 or/18-23 (7842)

25 exp quality control/ (249883)

26 exp standard/ (340449)

27 exp checklist/ (5144)

28 (quality or standard* or criteri* or characteristic* or guideline* or guidance* or checklist* or check list* or score$1 or scoring).tw. (5398001)

29 (adher* or comply$ or compli$2 or complian* or conform* or evaluat* or assess*).tw. (7177120)

30 or/25-29 (10732561)

31 24 and 30 (4298)

32 limit 31 to yr="1990 -Current" (4263)

33 32 use emez (3590)

34 17 or 32 (11916)

35 limit 34 to yr="2009-2012" (5459)

36 remove duplicates from 35 (4830)

37 limit 34 to yr="2000-2008" (4774)

38 remove duplicates from 37 (4370)

39 limit 34 to yr="1990-1999" (1683)

40 remove duplicates from 39 (1624)

41 36 or 38 or 40 (10824)

42 41 use prmz (8160) MEDLINE records

43 41 use emez (2664) Embase records

**CLIB:**

| **ID** | **Search** | **Hits** |
| --- | --- | --- |
| #1 | [MeSH descriptor **Meta-Analysis as Topic** explode all trees](http://onlinelibrary.wiley.com/o/cochrane/searchHistory?mode=runquery&qnum=1) | 481 |
| #2 | [MeSH descriptor **Review Literature as Topic** explode all trees](http://onlinelibrary.wiley.com/o/cochrane/searchHistory?mode=runquery&qnum=2) | 93 |
| #3 | [((review* or overview*) NEAR/2 synthes*):ti,ab,kw](http://onlinelibrary.wiley.com/o/cochrane/searchHistory?mode=runquery&qnum=3) | 46 |
| #4 | [(umbrella review* or meta-review* or metareview*):ti,ab,kw](http://onlinelibrary.wiley.com/o/cochrane/searchHistory?mode=runquery&qnum=4) | 18 |
| #5 | [("review of reviews" or "reviews of reviews" or "overview of reviews" or "overviews of reviews" or "overview of overviews" or "overviews of overviews"):ti,ab,kw](http://onlinelibrary.wiley.com/o/cochrane/searchHistory?mode=runquery&qnum=5) | 34 |
| #6 | [("review of systematic reviews" or "reviews of systematic reviews" or "review of systematic overviews" or "reviews of systematic overviews" or "overview of systematic reviews" or "overviews of systematic reviews" or "systematic review of systematic reviews" or "systematic reviews of systematic reviews"):ti,ab,kw](http://onlinelibrary.wiley.com/o/cochrane/searchHistory?mode=runquery&qnum=6) | 25 |
| #7 | [(#1 OR #2 OR #3 OR #4 OR #5 OR #6)](http://onlinelibrary.wiley.com/o/cochrane/searchHistory?mode=runquery&qnum=7) | 673 |
| #8 | [MeSH descriptor **Quality Control** explode all trees](http://onlinelibrary.wiley.com/o/cochrane/searchHistory?mode=runquery&qnum=8) | 449 |
| #9 | [MeSH descriptor **Guideline Adherence** explode all trees](http://onlinelibrary.wiley.com/o/cochrane/searchHistory?mode=runquery&qnum=9) | 521 |
| #10 | [MeSH descriptor **Checklist** explode all trees](http://onlinelibrary.wiley.com/o/cochrane/searchHistory?mode=runquery&qnum=10) | 27 |
| #11 | [Any MeSH descriptor with qualifier: **ST**](http://onlinelibrary.wiley.com/o/cochrane/searchHistory?mode=runquery&qnum=11) | 9776 |
| #12 | [(quality or standard* or criteri* or characteristic* or guideline* or guidance* or checklist* or score$1 or scoring):ti,ab,kw](http://onlinelibrary.wiley.com/o/cochrane/searchHistory?mode=runquery&qnum=12) | 175370 |
| #13 | [(adher* or comply$ or compli$2 or complian* or conform* or evaluat* or assess*):ti,ab,kw](http://onlinelibrary.wiley.com/o/cochrane/searchHistory?mode=runquery&qnum=13) | 268564 |
| #14 | [(#8 OR #9 OR #10 OR #11 OR #12 OR #13)](http://onlinelibrary.wiley.com/o/cochrane/searchHistory?mode=runquery&qnum=14) | 337123 |
| #15 | [(#7 AND #14)](http://onlinelibrary.wiley.com/o/cochrane/searchHistory?mode=runquery&qnum=15) | 335 |
| #16 | [(#15), from 1990 to 2012](http://onlinelibrary.wiley.com/o/cochrane/searchHistory?mode=runquery&qnum=16) | 323 |

DSR - 37

DARE – 75

Methods - 62

HTA – 13

*Records from CENTRAL and NHS EED not downloaded*
