# Supplementary material for: Identifying approaches for assessing methodological and reporting quality of systematic reviews: a descriptive study
Source: Syst Rev. 2017 Jun 19;6:117. doi: 10.1186/s13643-017-0507-6 (PMC5477124; doi:10.1186/s13643-017-0507-6)
Supplement: Supplementary file 3 — List of included studies [33, 40, 42–51, 63–126]. (DOCX 22 kb) [file 13643_2017_507_MOESM3_ESM.docx]

**Additional File 3. List of Included Studies**

| **Study** | **Title** |
| --- | --- |
| Al Faleh 2009 ^63^ | Reporting and methodologic quality of Cochrane Neonatal review group systematic reviews |
| Anttila 2012 ^64^ | Quality of evidence of assistive technology interventions for people with disability: An overview of systematic reviews |
| Assendelft 1995 ^45^ | The relationship between methodological quality and conclusions in reviews of spinal manipulation |
| Aziz 2013 ^65^ | Methodological quality and descriptive characteristics of prosthodontic-related systematic reviews |
| Barbosa 2012 ^66^ | Neuraxial anesthesia compared to general anesthesia for procedures on the lower half of the body: systematic review of systematic reviews |
| Barton 2008 ^46^ | Evaluation of the scope and quality of systematic reviews on nonpharmacological conservative treatment for patellofemoral pain syndrome |
| Biondi-Zoccai 2006 ^67^ | Compliance with QUOROM and quality of reporting of overlapping meta-analyses on the role of acetylcysteine in the prevention of contrast associated nephropathy: case study |
| Boluyt 2007 ^68^ | State of the evidence on acute asthma management in children: a critical appraisal of systematic reviews |
| Braga 2011 ^69^ | Methodological concerns and quality appraisal of contemporary systematic reviews and meta-analyses in pediatric urology |
| Brito 2013 ^70^ | Systematic reviews supporting practice guideline recommendations lack protection against bias |
| Canter 2005 ^71^ | Sources of bias in reviews of spinal manipulation for back pain |
| Choi 2001 ^72^ | Examining the evidence in anesthesia literature: a critical appraisal of systematic reviews |
| Collier 2006 ^73^ | Cochrane Skin Group systematic reviews are more methodologically rigorous than other systematic reviews in dermatology |
| Conway 2013 ^74^ | Not all systematic reviews are systematic: a meta-review of the quality of systematic reviews for non-invasive remote monitoring in heart failure |
| de Bot 2011 ^75^ | Sublingual immunotherapy in children with allergic rhinitis: quality of systematic reviews |
| Delaney 2007 ^76^ | The quality of reports of critical care meta-analyses in the Cochrane Database of Systematic Reviews: an independent appraisal |
| Derry 2006 ^77^ | Systematic review of systematic reviews of acupuncture published 1996-2005 |
| Elangovan 2012 ^78^ | Quality Assessment of Systematic Reviews on Periodontal Regeneration in Humans |
| Fleming 2014 ^79^ | Systematic reviews published in higher impact clinical journals were of higher quality |
| Fleming 2013 ^80^ | A PRISMA assessment of the reporting quality of systematic reviews in orthodontics |
| Fleming 2013b ^81^ | Cochrane and non-Cochrane systematic reviews in leading orthodontic journals: a quality paradigm? |
| Gagnier 2013 ^82^ | Reporting and methodological quality of systematic reviews in the orthopaedic literature |
| Gebel 2007 ^83^ | The physical environment and physical activity: a critical appraisal of review articles |
| Glenny 2003 ^84^ | The assessment of systematic reviews in dentistry |
| Hardern 1996 ^44^ | Reviews in accident and emergency medicine: the past and the future |
| Hu 2011 ^85^ | Cochrane systematic reviews of Chinese herbal medicines: an overview |
| Jadad 1998 ^86^ | Methodology and reports of systematic reviews and meta-analyses: a comparison of Cochrane reviews with articles published in paper-based journals |
| Jadad 2000 ^87^ | Systematic reviews and meta-analyses on treatment of asthma: critical evaluation |
| Junhua 2007 ^88^ | Methodology and reporting quality of systematic review/meta-analysis of traditional Chinese medicine |
| Kelly 2001 ^89^ | Evaluating the quality of systematic reviews in the emergency medicine literature |
| Kitsiou 2013 ^90^ | Systematic reviews and meta-analyses of home telemonitoring interventions for patients with chronic diseases: a critical assessment of their methodological quality |
| Knox 2009 ^51^ | A review of the methodological features of systematic reviews in fetal medicine |
| Kuukasjarvi 2006 ^91^ | Overview of systematic reviews on invasive treatment of stable coronary artery disease |
| Latthe 2008 ^92^ | Nonsurgical treatment of stress urinary incontinence (SUI): grading of evidence in systematic reviews |
| Lawson 2005 ^93^ | Systematic reviews involving complementary and alternative medicine interventions had higher quality of reporting than conventional medicine reviews |
| Lee 2011 ^94^ | Qigong for healthcare: an overview of systematic reviews |
| Li 2014 ^95^ | Quality of reporting of systematic reviews published in "evidence-based" Chinese journals |
| Linde 2003 ^96^ | Characteristics and quality of systematic reviews of acupuncture, herbal medicines, and homeopathy |
| Lundh 2009 ^97^ | Quality of systematic reviews in pediatric oncology--a systematic review |
| Luo 2014 ^98^ | Oral Chinese proprietary medicine for angina pectoris: An overview of systematic reviews/meta-analyses |
| Ma 2011 ^99^ | Epidemiology, quality and reporting characteristics of systematic reviews of traditional Chinese medicine interventions published in Chinese journals |
| Ma 2012 ^100^ | Epidemiology, quality, and reporting characteristics of systematic reviews of acupuncture interventions published in Chinese journals |
| MacDonald 2010 ^101^ | Assessment of the methodological quality of systematic reviews published in the urological literature from 1998 to 2008 |
| McAlister 1999 ^50^ | The medical review article revisited: has the science improved? |
| McGee 2013 ^102^ | Systematic reviews of surgical procedures in children: quantity, coverage and quality |
| Melchiors 2012 ^103^ | An analysis of quality of systematic reviews on pharmacist health interventions |
| Moher 2002 ^104^ | Assessing the quality of reports of systematic reviews in pediatric complementary and alternative medicine |
| Moher 2007 ^33^ | Epidemiology and reporting characteristics of systematic reviews |
| Momeni 2013 ^105^ | The quality of systematic reviews in hand surgery: an analysis using AMSTAR |
| Moseley 2009 ^106^ | Cochrane reviews used more rigorous methods than non-Cochrane reviews: survey of systematic reviews in physiotherapy |
| Mrkobrada 2008 ^107^ | Need for quality improvement in renal systematic reviews |
| Nicolau 2013 ^108^ | Methodological and reporting quality of systematic reviews on tuberculosis |
| Olsen 2001 ^109^ | Quality of Cochrane reviews: assessment of sample from 1998 |
| Padula 2012 ^110^ | Analysis of reporting of systematic reviews in physical therapy published in Portuguese |
| Panic 2013 ^111^ | Evaluation of the endorsement of the preferred reporting items for systematic reviews and meta-analysis (PRISMA) statement on the quality of published systematic review and meta-analyses |
| Papageorgiou 2011 ^112^ | Evaluation of methodology and quality characteristics of systematic reviews in orthodontics |
| Pieper 2014 ^47^ | Impact of choice of quality appraisal tool for systematic reviews in overviews |
| Pieper 2013 ^113^ | State of evidence on the relationship between high-volume hospitals and outcomes in surgery: A systematic review of systematic reviews |
| Remschmidt 2014 ^114^ | Methodological quality of systematic reviews on influenza vaccination |
| Santaguida 2011 ^115^ | Systematic reviews identify important methodological flaws in stroke rehabilitation therapy primary studies: review of reviews |
| Schmitter 2013 ^116^ | A flood tide of systematic reviews on endodontic posts: methodological assessment using of R-AMSTAR |
| Seo 2012 ^117^ | Quality assessment of systematic reviews or meta-analyses of nursing interventions conducted by Korean reviewers |
| Shea 2006a ^118^ | Does updating improve the methodological and reporting quality of systematic reviews? |
| Shea 2006b ^119^ | Scope for improvement in the quality of reporting of systematic reviews. From the Cochrane Musculoskeletal Group |
| Shea 2002 ^120^ | A comparison of the quality of Cochrane reviews and systematic reviews published in paper-based journals |
| Sheikh 2007 ^40^ | A review of the methodological features of systematic reviews in maternal medicine |
| Silagy 1993 ^121^ | An analysis of review articles published in primary care journals |
| Smith 1997 ^43^ | An analysis of review articles published in four anaesthesia journals |
| Stroup 2001 ^42^ | Characteristics of meta-analyses related to acceptance for publication in a medical journal |
| Tunis 2013 ^122^ | Association of study quality with completeness of reporting: have completeness of reporting and quality of systematic reviews and meta-analyses in major radiology journals changed since publication of the PRISMA statement? |
| Turner 2013 ^48^ | An evaluation of epidemiological and reporting characteristics of complementary and alternative medicine (CAM) systematic reviews (SRs) |
| Weed 2011 ^123^ | Quality of reviews on sugar-sweetened beverages and health outcomes: a systematic review. [Review] |
| Weir 2012 ^49^ | Reviewing the impact of computerized provider order entry on clinical outcomes: The quality of systematic reviews |
| Wen 2008 ^124^ | The reporting quality of meta-analyses improves: a random sampling study |
| Windsor 2012 ^125^ | Methodological quality of systematic reviews in subfertility: a comparison of Cochrane and non-Cochrane systematic reviews in assisted reproductive technologies |
| Xu 2012 ^126^ | Quality assessment for systematic review /meta-analysis on antidepressant therapy published in chinese journals |
